# Room Temperature Ferrimagnetism and Ferroelectricity in Strained, Thin Films of BiFe_0.5_Mn_0.5_O_3_

**DOI:** 10.1002/adfm.201401464

**Published:** 2014-10-14

**Authors:** Eun-Mi Choi, Thomas Fix, Ahmed Kursumovic, Christy J Kinane, Darío Arena, Suman-Lata Sahonta, Zhenxing Bi, Jie Xiong, Li Yan, Jun-Sik Lee, Haiyan Wang, Sean Langridge, Young-Min Kim, Albina Y Borisevich, Ian MacLaren, Quentin M Ramasse, Mark G Blamire, Quanxi Jia, Judith L MacManus-Driscoll

**Affiliations:** Department of Materials Science, University of Cambridge27 Charles Babbage Road, Cambridge, CB3 0FS, UK E-mail: emc63@cam.ac.uk; ISIS, Science and Technology Facilities Council, Rutherford Appleton LaboratoryDidcot, OX11 0QX, UK; National Synchrotron Light Source, Brookhaven National LaboratoryUpton, New York, 11973, USA; Center for Integrated Nanotechnologies, Los Alamos National LaboratoryLos Alamos, New Mexico, 87545, USA; Stanford Synchrotron Radiation Lightsource, SLAC National Accelerator LaboratoryMenlo Park, California, 94025, USA; Department of Electrical and Computer Engineering, Texas A&M UniversityCollege Station, TX, 77843–3128, USA; Materials Science and Technology Division, Oak Ridge National LaboratoryOak Ridge, Tennessee, 37831, USA; Division of Electron Microscopic Research, Korea Basic Science InstituteDaejeon, 305–806, Republic of Korea; SUPA School of Physics and Astronomy, University of GlasgowGlasgow, G12 8QQ, UK; SuperSTEM, SciTech DaresburyKeckwick Lane, Warrington, WA4 4AD, UK

**Keywords:** multiferroic, ferrimagnetism, ferroelectric, BiFeO_3_, BiMnO_3_

## Abstract

Highly strained films of BiFe_0.5_Mn_0.5_O_3_ (BFMO) grown at very low rates by pulsed laser deposition were demonstrated to exhibit both ferrimagnetism and ferroelectricity at room temperature and above. Magnetisation measurements demonstrated ferrimagnetism (*T_C_* ∼ 600K), with a room temperature saturation moment (*M_S_*) of up to 90 emu/cc (∼ 0.58 *μ_B_*/f.u) on high quality (001) SrTiO_3_. X-ray magnetic circular dichroism showed that the ferrimagnetism arose from antiferromagnetically coupled Fe^3+^ and Mn^3+^. While scanning transmission electron microscope studies showed there was no long range ordering of Fe and Mn, the magnetic properties were found to be strongly dependent on the strain state in the films. The magnetism is explained to arise from one of three possible mechanisms with Bi polarization playing a key role. A signature of room temperature ferroelectricity in the films was measured by piezoresponse force microscopy and was confirmed using angular dark field scanning transmission electron microscopy. The demonstration of strain induced, high temperature multiferroism is a promising development for future spintronic and memory applications at room temperature and above.

## 1. Introduction

In multiferroic materials, magnetism and ferroelectricity coexist, and if they are coupled they give rise to magnetoelectricity (ME) (or ferroelectromagnetism). Since most ferromagnets are conductive (with partially filled *d*-bands) and ferroelectrics are insulating (with filled bands), ferromagnetic ferroelectrics are scarce and currently non-existent at room temperature.[1] Multiferroic materials must not have either time reversal or spatial inversion symmetry which means there is only a small class of materials with both symmetries, typically those with frustrated magnetism, resulting from competing spin interactions. Room temperature multiferroics are currently the subject of intense scientific research because of various novel technological applications including magnetoelectric random access memory (MERAM), tunable multifunctional spintronic devices including four-state memory devices, and spin filters.[2–4] However, to date there have been no clear demonstrations of both room temperature (RT) ferroelectricity (FE) and strong ferro/ferrimagnetism (FM/FIM).

Among single-phase multiferroic materials, BiMnO_3_ (BMO) and BiFeO_3_ (BFO) have been extensively investigated. BMO is a rare multiferroic material which is FM (saturation moment, *M_S_*, of 3.6 *μ_B_*/f.u). While the FE transition temperature is relatively high at 400 K, the FM transition temperature (*T_C_*) is far below RT (*T_C_* ∼ 105 K), and the polarization is small (0.1 μC/cm^2^ at ∼90K).[5] BMO shows a weak negative magnetodielectric effect (MDE) near *T_C_* and exhibits a large gap between the FE and FM transition temperature.[6] To achieve practical ME applications of BMO, La-substitution on the *A*-site has been studied.[7] However, despite the enhancement of the ME effect, the *T_C_* remains low and the piezoresponse amplitude (*d_33_*) of 2 pm/V is lower than BFO films (60 pm/V).[7,8]

BFO has an incommensurate *G*-type antiferromagnetic (AFM) structure (*T_N_* ∼ 650 K) and ferroelectricity (*T_C_* ∼ 1103 K) caused by 6*s^2^* lone pair distortions of Bi^3+^ at the *A*-site.[9] In spite of the high ordering transition temperature of BFO, there are drawbacks for practical applications, most notably a weak magnetic moment due to the cycloidally modulated G-type AFM structure with a large period of 62 nm.[10]

With the aim of enhancing the magnetic moment in BFO while maintaining FE, various approaches have been followed including attempting to induce a local spin configuration in a *B*-site ordered structure, or creating a complex chiral or canted spin structure via substitution of different *3d* transition metal cations (transition metal, *TM* = Cr, Mn, Co, Ni, and Cu) at the *B*-sites.[11,12] The highest moment values achieved were for *TM* = Co, where 6 emu/cc was obtained at 10 K.[12] As far as Mn doping of the *B*-site goes, even though the theoretical predictions of Pálová et al. suggested that it may be possible to achieve co-existent FM and FE, in both bulk and thin films over a wide doping range only low moments have been observed.[10–23] However, in these earlier studies relatively thick films (>100 nm) were studied which were not highly strained.[19–22]

The hypothesis behind this work is that it may be possible to achieve a magnetic FE system in BiFe_0.5_Mn_0.5_O_3_ (BFMO) at RT by strain engineering. Recent reports in oxide systems have demonstrated that the magnetic state can be engineered by epitaxial strain.[24–26] Earlier, we showed that 30 nm films of BFMO grown on (001) SrTiO_3_ (STO) have a highly strained, coherent structure with a 2.5% higher *c* parameter than in high pressure bulk material and a high magnetic transition temperature (∼600 K) and saturation magnetic moment (90 emu/cc, 0.58 *μ_B_*/f.u).[27] However, details of the strain effect, the elucidation of precise magnetic structure and the measurement of ferroelectric properties were not determined previously.

**Scheme 1:**
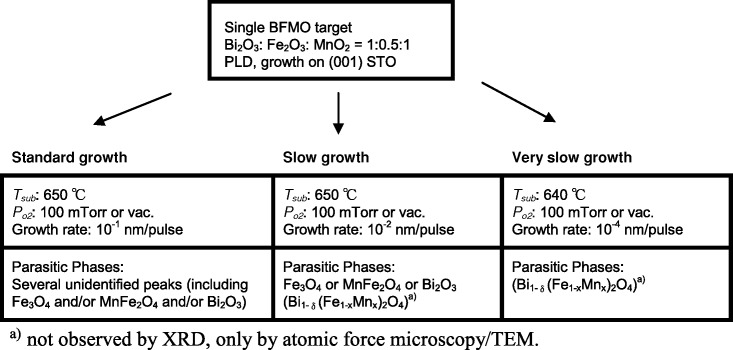
Summary of the target fabrication and film growth conditions explored and phases obtained.

In this work, around 60 growth runs were undertaken to elucidate the optimum conditions for achieving high quality epitaxy and high magnetic moment (a summary of the range of growth conditions are shown in **Scheme** **1**. In addition, we tried to grow BFMO films on various substrates, (001) LaAlO_3_, (001) yttria-stabilized zirconia, and (111) STO with different in-plane lattice mismatch (3 – 9%). For all these substrates, the BFMO perovskite phase was not well stabilized and many impurity phases formed. The Bi-Fe-Mn-O is a very complex system because of the possible mixed Mn and Fe valences and because there are a number of different possible phases.[28] Hence, we found that the BFMO perovskite phase only forms if there is a close lattice match with the substrate (i.e., (001) STO), and if the films are very thin and highly strained. Meticulous growth control is required to achieve clean, highly epitaxial, strained BFMO (**Table** **1**).

**Table 1:** Table of samples representative of the range of very slow growth conditions (for optimized films), as well as standard and slow growth conditions, together with saturation magnetic moment values measured and magnetic measurements performed

Film no.	Growth rate [nm/pulse]	T_S_ [°C]	Substrate (FWHM of *Δω*)	Thickness [nm]	Magnetic moment at RT and 3 kOe [emu/cc]	Measurements
**Very slow growth rate**						
1	10^−4^	640	(001)STO (0.019)	30	90	SQUID, VSM, XMCD, PNR, EELS
2	10^−4^	640	(001)Nb:STO (0.023)	33	60	SQUID, VSM, PFM
3	10^−4^	640	(001)Nb:STO (0.022)	70	50	SQUID, VSM
**Standard and slow growth rate**						
Many films	10^−1^	600	(001)STO	40–300	0	VSM
4	10^−2^	650	(001)STO (0.020)	120	10	SQUID, VSM
5	10^−2^	650	(001)STO	30	0a)	VSM

a) Film5 shows a pyramidal structure of spinel Bi_1-δ_(Mn_1-x_Fe_x_)_2_O_4_ in atomic force microscopy/TEM. Only a diamagnetic background from the substrate is observed in magnetic measurement.

## 2. Results and Discussion

As shown in Scheme 1 and Table 1, it was necessary to use ‘very slow’ growth rates to achieve near-phase pure BFMO with strong magnetic moments. Faster grown films contained several impurity phases and weak magnetic signals. As observed by transmission electron microscopy (TEM) and AFM, all the ‘very slow’ growth films contained only one second phase, Bi_1-δ_(Mn_1-x_Fe_x_)_2_O_4_, which had a spinel structure. The phase was strained to the (001) BFMO so that the (111) Bi_1-δ_(Mn_1-x_Fe_x_)_2_O_4_ peak overlapped with the (002) BFMO peak. The Bi_1-δ_(Mn_1-x_Fe_x_)_2_O_4_ phase was always observed at the grain boundaries of the BFMO towards the surface of the film, which indicates that it forms as a result of Bi-evaporation from the BFMO grain boundaries during growth. Since the growth temperature must be low to avoid excessive Bi loss during growth, the growth rate also needs to be low to give sufficient kinetics to achieve the required epitaxy (it is clear from Figure 2 below that very high epitaxial quality and coherent strain are required to give strong magnetism). Of course, slow growth will also mean some Bi loss, and so it is a matter of very finely balancing BFMO phase stabilization plus epitaxial quality, versus Bi loss.

The likely reason that Fe_3­_O_4_ + MnFe_2_O_4_ + Bi_2_O_3_ phases were found in the fast grown films and not in the slower grown films is that under fast growth conditions, the thermodynamically preferred perovskite BFMO phase (stabilized through epitaxy with the perovskite substrate) is kinetically not preferred: here the growth is too fast to allow the complex composition to crystallize sufficiently. Hence, the simpler binary and ternary component oxides are kinetically stabilized.

It was absolutely necessary to be certain that the magnetism did not originate from Bi_1-δ_(Mn_1-x_Fe_x_)_2_O_4_. Hence, we measured the properties of many ‘slow growth’ films where the phase was observed to be present at a level of up to 20% (as determined from AFM and TEM). We found that there was no ‘slow growth’ film which contained *only* BFMO+ Bi_1-δ_(Mn_1-x_Fe_x_)_2_O_4_ and which had a moment above 10 emu/cc. For example, Film5 (Table 1) contained at least 20% of Bi_1-δ_(Mn_1-x_Fe_x_)_2_O_4_ (as seen in TEM and AFM), but only a diamagnetic signal from the STO substrate was obtained. Hence, it was certain that Bi_1-δ_(Mn_1-x_Fe_x_)_2_O_4_ was non-magnetic. Therefore, the magnetic signals in the ‘very slow growth’ films (where only Bi_1-δ_(Mn_1-x_Fe_x_)_2_O_4_ was observed in addition to the BFMO film) were *intrinsic* to highly strained BFMO.

**Figure 1:**
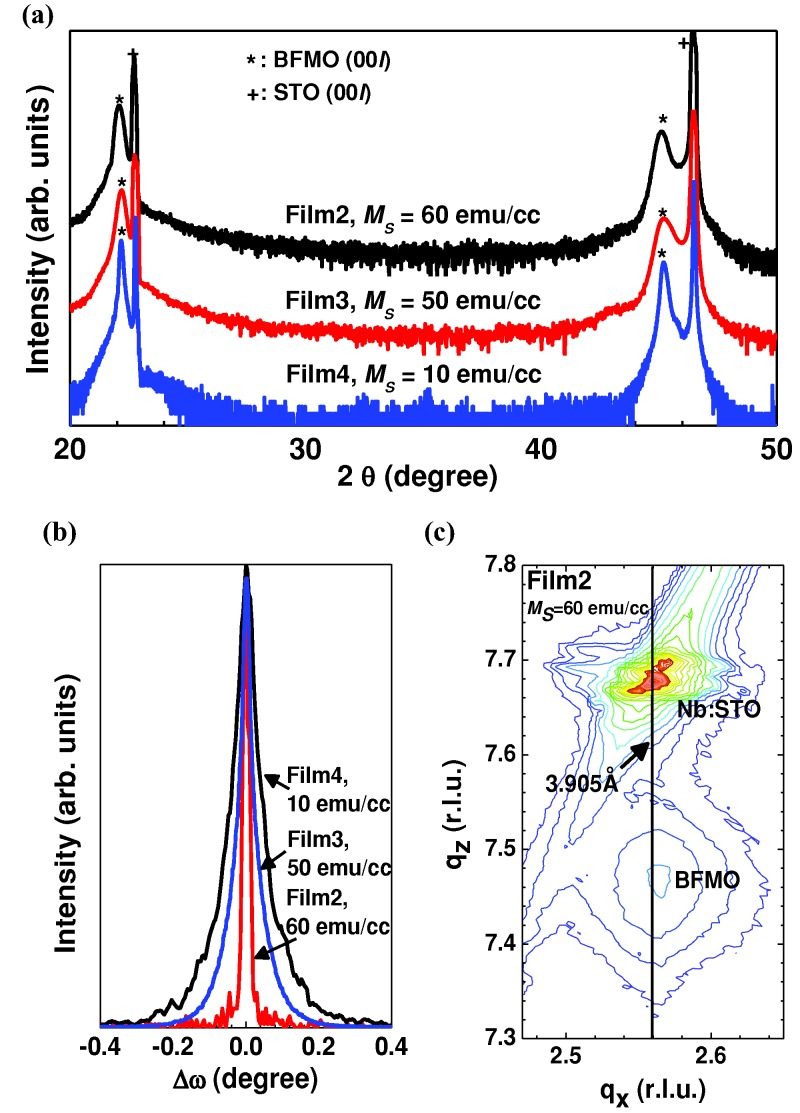
Comparison of thin and thick BFMO films: a) X-ray diffraction (XRD) spectra (*θ – 2θ* scan) for Film 2, 3 and 4. b) *ω*-rocking curves of the (002) diffraction peaks of Film2 (30 nm thick), Film3 (60 nm thick) and Film4 (120 nm thick). c) Reciprocal space map (RSM) around the (103) reflections of Nb:STO of Film2.

In the X-ray diffraction (XRD) spectra of **Figure** **1**a, we show highly epitaxial growth with sharp (00*l*) peaks of BFMO to the left of the (00l) STO peaks. The *ω*-rocking curve of Figure 1b shows that the more highly crystalline films have the higher moments. Looking more closely at the best film (Film2, 30 nm thick) by X-ray reciprocal space mapping (RSMs) near the (103) STO peak and (103) BFMO peak (Figure 1c), we see epitaxial straining of the BFMO to the STO (indicated by the vertical line). The in-plane lattice parameter of the STO was measured to be 3.907 ± 0.002 Å. The out-of-plane and in-plane lattice parameters of Film2 were c = 4.015 ± 0.003 Å and a = 3.900 ± 0.019 Å. To further probe the details of the strained film structure, bright field TEM images were taken along the [100] zone axis of the Nb:STO substrate of Film2 (see Supplementary Note and Figure S1 online). In short, the film was found to be clean, stoichiometric in Fe and Mn (i.e., 1:1 ratio from energy dispersive X-ray spectroscopy in TEM within the error of 2–5%), highly epitaxial and coherently strained throughout the thickness. It is not expected that there will be coherent strain in films of thickness much greater than 30 nm.

**Figure 2:**
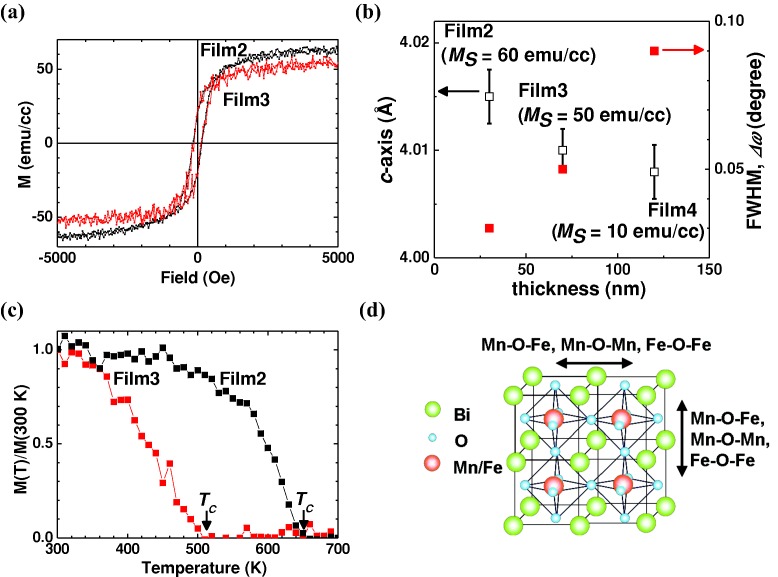
Strain effect on the magnetic properties of BFMO films: a) Magnetic hysteresis (*M – H*) curves in the range of -5 ≤ H ≤ 5 kOe at RT with the field applied in the sample plane for Film2 (black square) and Film3 (red square). b) The relationship between c-axis lattice parameter (open squares), FWHM of *ω-*rocking curves (red squares) and film thickness. c) Normalized in-plane *M – T* curves of Film2 and Film3. The magnetic transition temperatures of Film2 and Film3 are > 600 K and ∼500 K, respectively. d) Schematic illustration for disordered BiFe_0.5_Mn_0.5_O_3_ structure and various combinations of *B-O-B(B′)* bond.

**Figure** **2** demonstrates the strain effect on the magnetic properties of BFMO. As shown in Figure 2a, the films have maximum magnetic moments of 50–60 emu/cc (∼0.4 *μ_B_*/f.u) at 5 kOe at 300 K, which is just less than half of the theoretical 1 *μ_B_*/f.u. value for fully ordered double perovskite BFMO, indicating partial or full disorder of Fe and Mn.[24,29] We discuss this point more later on.

The need for excellent epitaxial growth and highly strained films is highlighted by comparing XRD and magnetic measurements. Figure 2b shows the relationship between the *c-*axis parameter and full width half maximum (FWHM) of the *ω*-rocking curves, and the saturation magnetic moment (*M_S_*) at 5 kOe: the thinner films are more highly strained, have higher c/a values, have better epitaxial quality, and have higher *M_S_* values. As shown from Table 1 it is clear that very high quality substrates (determined from the substrate FWHM values of *ω*-rocking curve of (002) peak (<0.02°) and a single sharp (103) peak (<0.03°)) are necessary to give strong magnetism, but this is not a necessary and sufficient condition, because if the films are grown fast or thick (>70 nm), the substrate epitaxial quality is not transferred to the film.

According to the trend of Figure 2b, films above ∼120 nm should have a negligible magnetic moment. Hence it is understandable why previously studied thicker films from the literature had very small or zero moments, e.g., a 180 nm thick film with a *c-*axis of 3.952 Å had an *M_S_* value of 4 emu/cc at RT and 1.5 T.[20] The magnetic moments for the most strained films of our study (<70 nm) are around an order of magnitude higher than literature values of thicker (>100 nm) BFMO films.[11,12,19–22] On the other hand, the values are around 30% lower than the best values (90 emu/cc) we obtained for ∼30 nm films on higher quality pure STO substrates (FWHM values of *ω*-rocking curve of (002) peak of 0.019° and (103) peak of 0.03°) where there is better epitaxy than on Nb:STO (FWHM values of *ω*-rocking curve of (002) peak of 0.023° and (103) peak of 0.10°), but it was necessary to use the Nb:STO conducting substrates in this work to measure FE properties.[27] The magnetic transition temperatures (*T_C_*) are much higher than bulk BMO (∼105 K). Film2 shows a sharp *T_C_* above 600 K and the less strained Film3 shows a reduced *T_C_* of 500 K (Figure 2c).

In order to determine whether there was any significant ordering of Fe and Mn atoms in the checkerboard fashion characteristic of a true double perovskite, careful studies were carried out using electron energy loss spectroscopy (EELS) spectrum imaging along the primitive [110] direction on Film2. No long range *chemical* ordering of Mn and Fe could be evidenced in these studies, and any such ordering, if present, must be very short range, in regions much smaller than the thickness of a typical scanning transmission electron microscope (STEM) sample (i.e., <<20 nm). A cation disordered perovskite structure with the possible metal oxygen bonds along the in-plane and out-of-plane directions is shown in Figure 2d. Studies of the ordering in the films did, however, demonstrate that there is distinct *structural* ordering in the form of a symmetry reduction, at least in parts of the upper layers of these films (See supplementary Note and Figure S2 online). This ordering consists of a displacive transformation resulting in the formation of the √2*a*_p_ × 4*a*_p_ × 2√2*a*_p_ (*a*_P_ is the parameter of the cubic perovskite subcell) orthorhombic *Pnma* phase previously reported by Belik.[30] All the evidence suggests that this *Pnma* phase is formed in such a way that domains with the [010] axis of the orthorhombic phase lie in plane, and this is expected, as this domain configuration would reduce misfit strain to the Nb:STO substrate.

To probe the origin of the magnetic properties, we investigated the valence states by X-ray magnetic circular dichroism (XMCD) and X-ray absorption spectroscopy (XAS, See Supporting Information Figure S3 online) for Film1. EELS was also carried out to investigate the chemical composition and valence states of Fe and Mn. XMCD measurements were performed at Mn and Fe *L*_2,3_-edges on Film1. XMCD provides, through the use of sum rules, access to the spin and orbital moments of the atomic level as well as element sensitivity.[31] Moreover, from the response of the XMCD signal as a function of the magnetic field, we could determine the spin directions.

**Figure 3:**
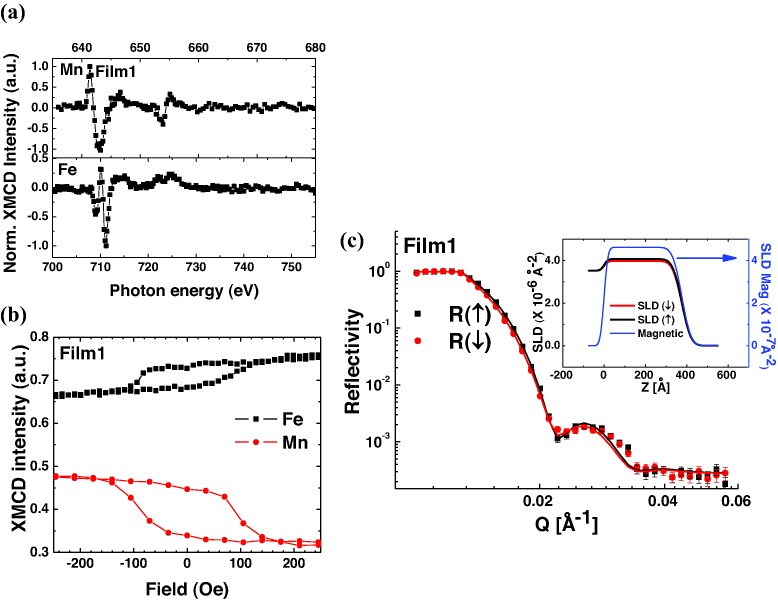
X-ray magnetic circular dichroism (XMCD) and spin polarized neutron reflectivity (PNR) for BFMO on STO (Film1): a) XMCD spectra of Film1 at the Fe *L_2,3_* edge (lower panel) and Mn *L_2,3_* edge (upper panel) at 300 K in total electron yield mode (TEY) after pulsing at 9.5 kOe for Film1. b) Fe (black) and Mn (red) *L_3_* edge XMCD (TEY) vs applied magnetic field at 300 K for Film1. c) Spin polarized neutron reflectivity (PNR) measured for Film1 at room temperature at an in-plane saturating field of 3 kOe. The inset shows the structural and magnetic SLD profile.

**Figure** **3**a and b show XMCD spectra at the Fe and Mn *L*_2,3_ edges and the magnetic field dependence at Fe and Mn *L*_3_-edges, respectively. These data confirm that Fe and Mn order antiferromagnetically with respect to one other, with the Fe magnetization parallel and the Mn antiparallel to the field. Hence the film is FIM. According to Goodenough-Kanamori (GK) rules, if the valences of Fe and Mn are Fe^3+^ (3*d_5_*, *t*^3^*_2g_e*^2^*_g_*) and Mn^3+^ (3*d_4_*, *t*^3^*_2g_e_1g_*), and the Fe and Mn cations are ordered, then there will be antiferromagnetic coupling, with FIM magnetization of 1 *μ_B_*/f.u.[18,32] In our films, there is no evidence of long range Fe^3+^ and Mn^3+^ ordering, i.e., we have the following possible bonds Fe^3+^-O-Fe^3+^, Mn^3+^-O-Mn^3+^ or Fe^3+^-O-Mn^3+^ (Figure 2d) which all favor AFM interactions. Since only the Fe^3+^-O-Mn^3+^ bond gives rise to an overall moment, the reduced moments measured of ≤90 emu/cc (0.58 *μ_B_*/f.u.) from the ordered 1 *μ_B_*/f.u. value are understood.[24,29]

Both XAS spectra of Fe and Mn exhibit a multiplet structure indicating mixed valence of Fe (2+/3+) and Mn (2+/3+) with a predominance of 3+. These mixed valences are consistent with the presence of some non-magnetic Bi_1-δ_(Mn_1-x_Fex)_2_O_4_ outgrowths at the film surface. The moment estimated by XMCD (0.13 *μ_B_*/f.u.) was lower than the superconducting quantum interference device (SQUID) measurement (90 emu/cc, 0.58 *μ_B_*/f.u.), likely because the XMCD penetration depth is only 5 – 10 nm, and so the signal is influenced by surface degradation effects and non-magnetic Bi_1-δ_(Mn_1-x_Fe_x_)_2_O_4_ outgrowths.[33]

Polarized neutron reflectivity (PNR) was undertaken after the SQUID measurements. PNR allows the extraction of the structural and magnetic depth profile in nanoscale systems.[34] The reflectivity is measured as a function of the spin eigenstate of the neutron being either parallel R (↑) or antiparallel R (↓) to a quantization axis defined by the applied magnetic field which is in the plane of the sample. The reflectivity, along with the model fit, is shown in Figure 3c. The substrate/film interface is relatively sharp (located at Z = 0 nm). From the scattering length density (SLD) it is clear that the magnetic induction of the BFMO is homogeneous throughout the film thickness, which is an important requirement for technological applications.

**Figure 4:**
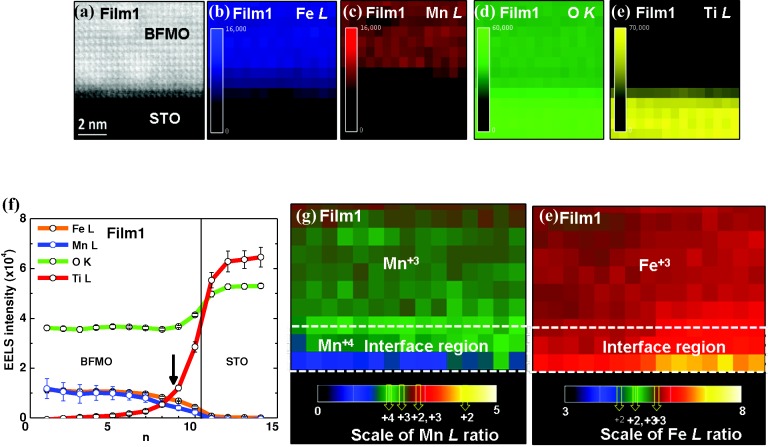
Electron energy loss spectroscopy (EELS) analysis of BFMO on STO (Film1): a) ADF STEM image of the sample area for the analysis. b–e) EELS intensity distributions of Fe *L*, Mn *L*, O *K*, and Ti *L* edges, respectively. f) Averaged profiles of the respective EELS signals across the interface (marked by solid line). g,h) Valence state maps of Mn and Fe at the part of BFMO film. The maps were obtained by EELS intensity ratios of the respective Mn and Fe *L* edges.

**Figures** **4**b–e show EELS maps of Fe *L*, Mn *L*, O *K*, and Ti *L* edges, for the corresponding annular dark field (ADF) STEM image (Figure 4a). In the BFMO film above the interface region, the maps reveal that the distribution of Fe^3+^ and Mn^3+^ is uniform (Figure 4f–h), i.e., Fe^3+^ and Mn^3+^ were observed well into the bulk of the film. At the interface region there is some Ti diffusion from the substrate into the film (to around 2 unit cells) which presumably leads to the Mn^4+^ near the interface. The volume of this region is too small to explain the magnetic moments observed.

Now we discuss the possible origin of the high *T_C_* FIM phase in disordered, highly strained, crystalline BFMO. First we note that transition metal perovskites show a great sensitivity of properties to bond angles and bond lengths.[18–21,24–26,35] As already mentioned, one of three strain mediated mechanisms can explain the behavior observed.

The first is that strain can affect the in-built molecular electric field which enhances the strength of superexchange interaction to minimize the magnetoelectric energy. This is more effective in Bi compounds because of the Bi 6*s^2^* loan pair than La-based ones, e.g. ordered FIM LaFe_0.5_Mn_0.5_O_3_ (LFMO).[24], [37] Indeed, despite having a disordered structure, strained thin films of BiCo_0.5_Mn_0.5_O_3_ (BCMO) have a very high FM *T_C_* of 800 K, which is 700 K higher than disordered polycrystalline BCMO (*T_C_* = 95 K).[24]

The second is the strain induced change of the *B*-O-*B*(*B*′) bond angle and bond length by *B*O_6_ octahedral rotation. The great sensitivity of magnetic properties to bond lengths and angles is well known in BMO.[36] Also, recently in ultra thin (<10 unit cell) La_0.67_Sr_0.33_MnO_3_ (LSMO) films, a significant enhancement of magnetic transition temperature up to 560 K was found and was explained by strain induced rotation of the *BO_6_* octahedra.[25]

The third is the coupling between electron spin and lattice via strain which can tune multiferroic order parameters. Recently, Lee et al. reported that substrate strain can control multiple order parameters to achieve FM (*T_C_* of ∼4.2 K)/FE (*T_C_* of ∼250 K) in EuTiO_3_ (ETO) thin films under tensile strain, whereas bulk EuTiO_3_ is AFM and paraelectric (PE).[26]

We note however that even though strongly enhanced magnetic properties have been achieved by strain engineering, none of the above mentioned compounds, ETO, BCMO, or LFMO show *both* FM/FIM and FE near to RT.

**Figure 5:**
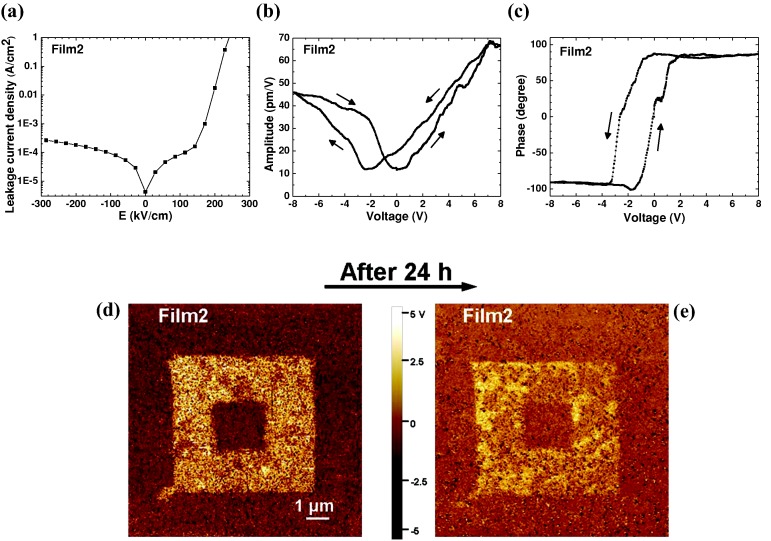
The ferroelectric properties of BFMO on Nb:STO (Film2): a) Leakage current density vs electric field of Film2 measured at RT where the positive contact is applied to the Pt electrode. b) Amplitude and c) phase of the PFM signal as a function of bias voltage. A slight shift towards negative electric field is caused by the use of different contacts on each side (Nb:STO and Pt). d,e) PFM phase contrast scan on Film2 after -20 V writing (10 × 10 μm^2^), +10V rewriting (6 × 6 μm^2^) and –20V rewriting (2 × 2 μm^2^) at RT before and after 24 h, respectively.

Finally, we turn to the FE properties of the films. Firstly the leakage current density vs electric field characteristics (*J – E*) were measured. As shown in **Figure** **5**a, an asymmetric *J – E* loop was observed with a sharp increase in the positive *E* – field region from 150 kV/cm when a positive voltage was applied on the Pt electrode. This originates from the difference in the electron injection from the top (Pt) and bottom (Nb:STO) electrodes which have different work functions (Pt: 5.3 eV and Nb:STO: 4.3 eV).[38] The level of leakage current lies between pure BFO and BMO thin films[13,38,39] and agrees with typical resistivity measurements of BFMO compared to BFO and BMO, namely 10^5^ versus 10^9^_­_(best reported for BFO, but possibly atypical) versus 10^2^ Ωcm for BMO.[26,40,41]

The piezoresponse force microscopy (PFM) amplitude and phase as a function of bias voltage (Figure 5b and c), while sweeping the DC voltage from +8 to -8 to +8V, show characteristic hysteretic behavior indicative of ferroelectricity at RT. Figure 5d and e show PFM images. The poling sequence was the following: a) application of a voltage of +10 V over a large area of 6 × 6 μm^2^ square, b) application of a voltage of -20 V over small square of size 2 × 2 μm^2^, c) reading the phase right after poling shown in Figure 5d, d) reading again after 24 h (Figure 5e). The piezoresponse phase can be reversibly switched by 180° (Figure 5d), and the phase contrast still remains after 24 h (Figure 5e). The piezoresponse amplitude of 45 pm/V is marginally lower than that of a pure BFO thin film with 50 – 60 pm/V[8] but higher than previously reported for 0.15% Mn-substituted BFO films (30 pm/V)[11] and La-substituted BMO films (2 pm/V).[7]

**Figure 6:**
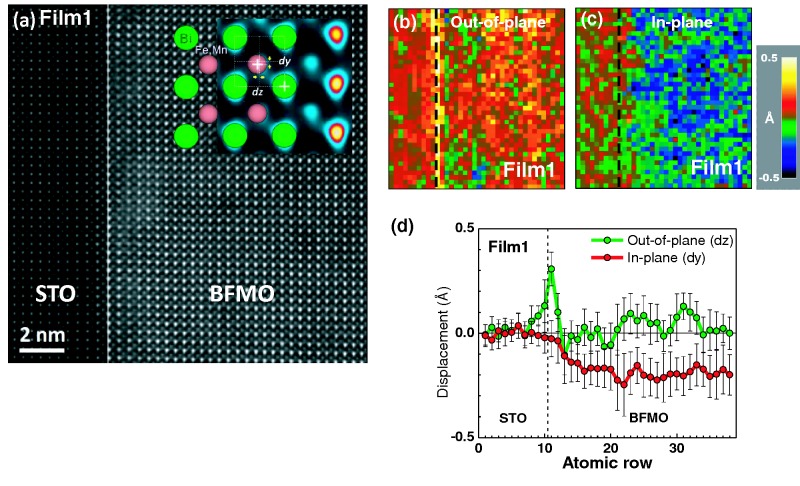
Polarization mapping by atom position quantification: a) Atomic resolution ADF STEM image of [100] oriented BFMO film on STO sample analyzed for polarization behavior. Inset: enlarged ADF STEM image in false color and atomic model to illustrate atom position quantification. b,c) Out-of-plane (*dz*) and in-plane (*dy*) displacement maps of B-site cations in BFMO thin film. d) Line profiles averaged over vertical rows of the maps (b – green and c-red). Dotted lines in each figure indicate the position of the interface between BFMO and STO substrate.

To directly decipher the polarization behavior of BFMO on a subunit-cell scale, we performed atom position quantification from aberration-corrected ADF STEM images of Film1 (**Figure** **6**a). This technique allows the mapping the relative cation displacements of the *B*-site atoms in the ABO_3_ perovskite structure with picometer precision.[42] A schematic of the quantification method to measure the relative displacements is given as inset in Figure 6a. Figure 6b and c show out-of-plane (*dz*, normal to the interface) and in-plane (*dy*, parallel to the interface) displacement maps of *B*-site cations in the structure, respectively; the profiles averaged over the vertical rows are plotted in Figure 6d. From these results, we can see that both the interface and the bulk of the film have characteristic polarization. At the interface, the out-of-plane (*dz*) displacements of *B*-site cations are sharply increased; thereafter, there is smaller displacement with some scatter as we move into film. In the bulk of the film, substantial negative (∼ –0.2 Å) *dy* displacements are detected. Displacements show substantial inhomogeneity as evidenced by error bars in Figure 6d. If we employ the approximation of a simple linear relationship between displacement and polarization, then high strain levels (Figure 1c and Figure S1c) and displacement of the *B*-site cations (Figure 6d) can explain the piezoresponse amplitude of BFMO along the out-of-plane direction.

## 3. Conclusion

In conclusion, we have demonstrated for the first time, coexistent ferrimagnetism and ferroelectricity at RT in BiFe_0.5_Mn_0.5_O_3_ (BFMO) by strain engineering. The most highly strained and crystalline films had a ferrimagnetic transition temperature of ∼600 K which is 500 K higher than bulk BMO and a piezoresponse amplitude of 45 pm/V. XMCD proved the ferrimagnetism in the films. Very slow growth rates and a narrow temperature window are critical for achieving good phase purity, very high crystallinity and coherent strain. There are three possible strain related mechanisms responsible for the high *T_C_*, with the Bi 6*s^2^* lone pair effect playing an important role. The work has opened up a new window for designing multiferroic materials through epitaxial strain control.

## 4. Experimental Section

*Film Fabrication*: Films were grown on (001) SrTiO_3_ (STO) and (001) Nb:STO substrates by pulsed laser deposition with a KrF laser. A mixed BFMO target was used for making BFMO. The BFMO target was prepared by thoroughly mixing stoichiometric amounts of high purity (99.99%) Bi_2_O_3_, Fe_2_O_3_, and MnO_2_, followed by sintering at 800 °C. For all the growth runs the film heater (infra red) temperature was set to 820°C but the actual temperature on the surface of surface was ∼180 °C lower than this, namely 640 °C. The laser pulse rate was 2 – 5 Hz. The oxygen pressure was fixed at 100 mTorr.

*Structural Analysis*: The phase and the crystalline quality of the thin films were investigated by *θ – 2θ* and asymmetric X-ray diffraction (XRD) on a Bruker D8 theta/theta diffractometer with Cu-*K_α_* radiation and a graded mirror. Reciprocal space maps (RSMs) were used to investigate the strain between the films and substrates. Transmission electron microscopy (TEM) was performed using a JEOL 4000EX microscope operating at 400 kV. Scanning transmission electron microscopy (STEM) was performed using a JEOL 4000EX microscope operating at 400 kV and two Nion UltraSTEMs, both operated at 100 kV and equipped with a Gatan Enfina EELS spectrometer, and a JEOL ARM200F (cold FEG) operated at 200 kV.

*Magnetic Analysis*: Magnetisation measurements (*M – T* and *M – H*) were performed using a Princeton vibrating sample magnetometer (VSM) and a superconducting quantum interference device (SQUID) magnetometer (Quantum Design, MPMS). The magnetic moment is converted from emu/cc to *μ_B_*/f.u. by using the volume of unit cell of BFMO measured by X-ray reflectivity and TEM. To investigate whether the magnetic origin in the films was FIM or FM, X-ray magnetic circular dichroism (XMCD) and X-ray absorption spectroscopy (XAS) were performed on beamline U4B at the National Synchrotron Light Source (NSLS), Brookhaven National Laboratory. The degree of circular polarization for the XMCD measurement was 70% with the incident photons aligned parallel to the magnetic field. The XMCD spectra were obtained by using a fixed circular polarization and switching the applied magnetic field by pulsing it to 9.5 kOe and measuring in remanence. The spectra were measured by total electron yield mode (TEY) at 300 K. XAS measurements were taken at an angle of 45° between the *E* – field of the X-rays and the out-of-plane direction of the sample. Polarized neutron reflectivity (PNR) measurements were performed using the CRISP reflectometer at ISIS[43] The measurements were carried out at RT in a field of 3 kOe applied in the plane of the sample perpendicular to the scattering direction. The fitting was done using the GenX reflectivity package.[44]

*Piezoresponse Measurements*: Leakage current density-voltage characteristics were measured using a Keithley 6487 picoammeter and a 2440 sourcemeter with a stabilization time of 10s. Nb:STO was used as the bottom electrode and for the top electrode; a 150 nm thick Pt pad of size 7.85 × 10^−5^ cm^2^ was deposited on the BFMO film. To characterize the ferroelectric properties, an atomic force microscope (Agilent SPM 5500) with 3 (MAC-3) lock-in amplifiers (LIAs) was used at RT in piezoresponse force microscopy (PFM) mode (out-of-plane signals). An Olympus Pt-coated tip (Asylum Research AC240TM) was used at the excitation frequency of 15 kHz with alternating current (AC) voltage *V_AC_* = 2.5 V, polarized by a direct current (DC) bias of *V_DC_* = ±10 to ±20 V. The piezoresponse signals were calibrated using a standard height (“Micro-Mesh”) sample and the LiNbO_3_ PFM factory standard sample (“Bruker”) with *d_33_* = 7.5 pm/V.

## Supporting Information

As a service to our authors and readers, this journal provides supporting information supplied by the authors. Such materials are peer reviewed and may be re-organized for online delivery, but are not copy-edited or typeset. Technical support issues arising from supporting information (other than missing files) should be addressed to the authors.
