# Supplementary material for: Room Temperature Ferrimagnetism and Ferroelectricity in Strained, Thin Films of BiFe0.5Mn0.5O3
Source: Adv Funct Mater. 2014 Oct 14;24(47):7478–87. doi: 10.1002/adfm.201401464 (PMC4511393; doi:10.1002/adfm.201401464)
Supplement: Supplementary file 1 — Supplementary [file adfm0024-7478-sd1.pdf]

Copyright WILEY-VCH Verlag GmbH & Co. KGaA, 69469 Weinheim, Germany, 2014.

ADVANCED FUNCTIONAL MATERIALS

Supporting Information

for *Adv. Funct. Mater.*, DOI: 10.1002/adfm.201401464

Room Temperature Ferrimagnetism and Ferroelectricity in Strained, Thin Films of $BiFe_{0.5}Mn_{0.5}O_3$

Eun-Mi Choi, Thomas Fix, Ahmed Kursumovic, Christy J. Kinane, Darío Arena, Suman-Lata Sahonta, Zhenxing Bi, Jie Xiong, Li Yan, Jun-Sik Lee, Haiyan Wang, Sean Langridge, Yong-Min Kim, Albina Y. Borisevich, Ian MacLaren, Quentin M. Ramasse, Mark G. Blamire, Quanxi Jia, and Judith L. MacManus-Driscoll*

Copyright WILEY-VCH Verlag GmbH & Co. KGaA, 69469 Weinheim, Germany, 2013.

Supporting Information

Room temperature Ferrimagnetism and Ferroelectricity in Strained, Thin Films of $BiFe_{0.5}Mn_{0.5}O_3$

Eun-Mi Choi, Thomas Fix, Ahmed Kursumovic, Christy J. Kinane, Darío Arena, Suman-Lata Sahonta, Zhenxing Bi, Jie Xiong, Lie Yan, Jun-Sik Lee, Haiyan Wang, Sean Langridge, Yong-Min Kim, Albina Y. Borisevich, Ian MacLaren, Quentin M. Ramasse, Mark G. Blamire, Quanxi Jia, and Judith L. MacManus-Driscoll*

Supplementary Note

Details of the strained film structure.

As shown **Figure S1a** and **b**, Film2 has an abrupt interface with the substrate, with a relatively smooth surface and homogeneous morphology. The upper inset of Figure S1b shows that the lattice has c/a ≈ 1.03 measured by XRD. **Figure S1c** shows a strain map along <110> calculated by making the 220 reflection in the Fourier transform and measuring the variations in lattice fringe spacing from the inverse Fourier transform image. The map shows a coherently strained film through the thickness with a localised region of tension only right at the interface (shown in blue).

Ordering in the Double perovskite structure.

There was significant evidence from STEM studies of a *Pnma structural* ordering of the film. This is summarized in **Figure S2**. This shows [010] and [201] patterns of the orthorhombic structure extracted as Fast Fourier Transforms from two different regions of an atomic resolution HAADF image along a pseudocubic <100> direction, and [211] and [2,-1,1] patterns of the orthorhombic structure from a scanning diffraction dataset recorded along a <110> pseudocubic direction. All patterns are compared to simulated diffraction patterns calculated for the *Pnma* structure described by Belik using the JEMS software package [1].

Supplementary References

[1] A. A. Belik, *Inorg. Chem*. **2013**, *52*, 2015.

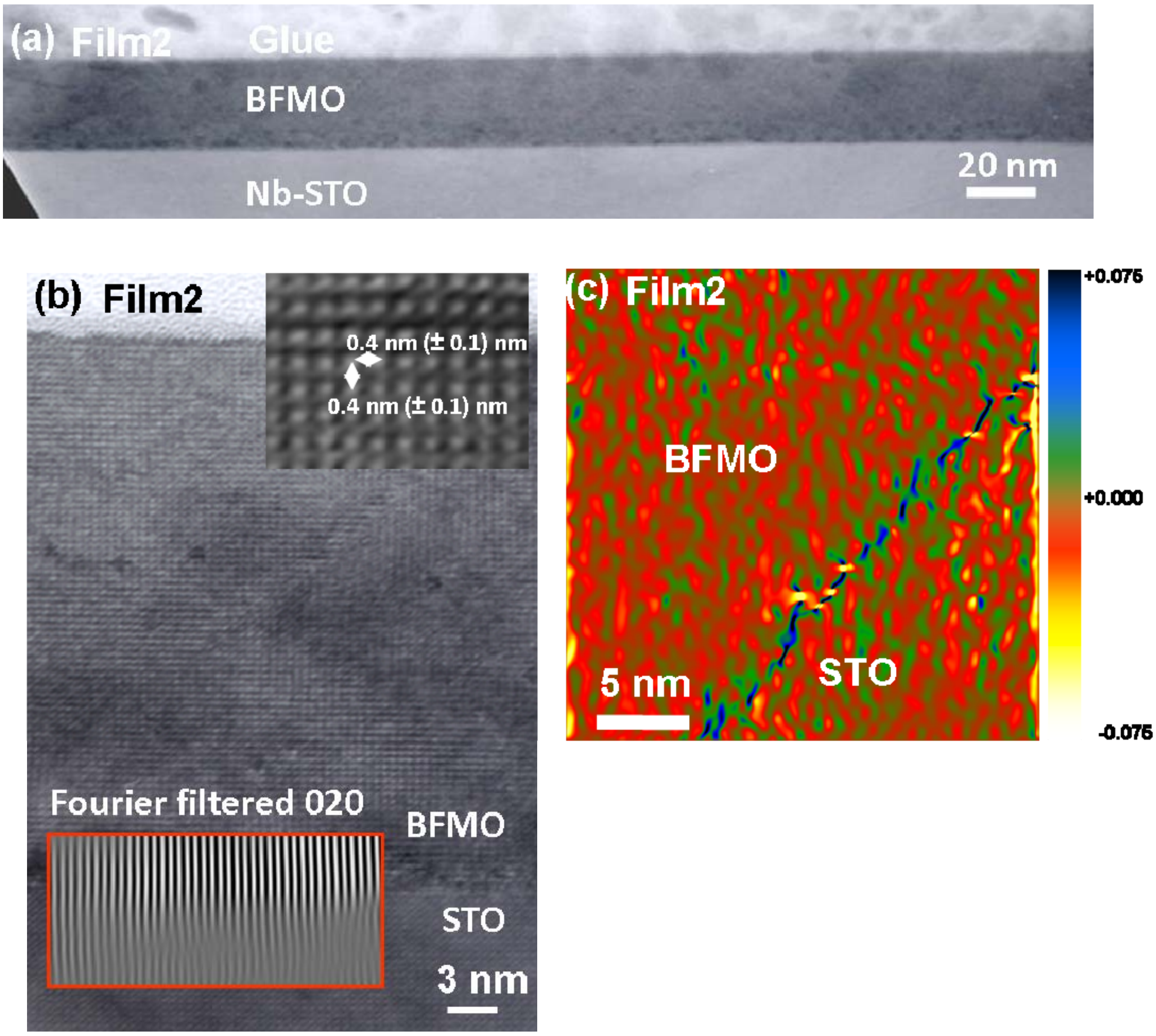

Figure S1. Details of the strained film structure: (a) TEM image of Film2 along the [100] zone axis of the Nb:STO (001) substrate. (b) High-resolution TEM. Upper inset shows measurements of lattice spacing. Lower inset shows Fourier filtered image of interface region. (c) Strain map along (110) from the (220) reflection.

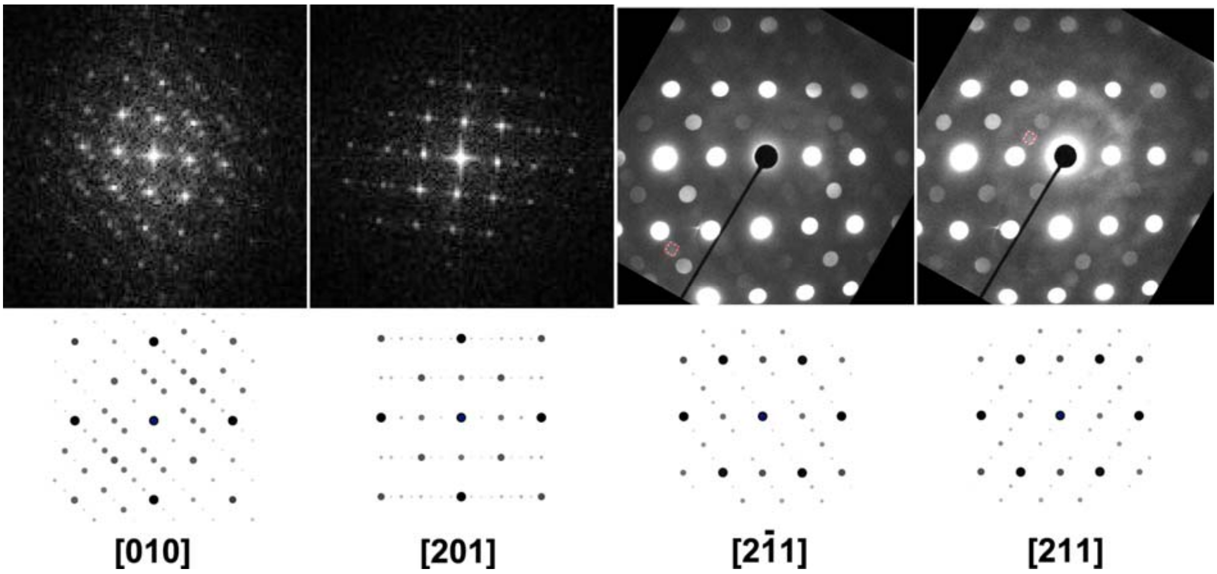

Figure S2. Diffraction patterns and Fast Fourier Transforms (FFTs) demonstrating the *Pnma structural* ordering of the film (Film2), by comparison to simulated electron diffraction patterns for the *Pnma* phase of Belik [1]. The [010] and [201] patterns are FFTs calculated from a HRSTEM image along the <001> direction of the primitive perovskite structure. The [2$\bar{1}$1] and [211] patterns are from a scanned diffraction dataset from a sample aligned along a <011> direction of the primitive perovskite structure.

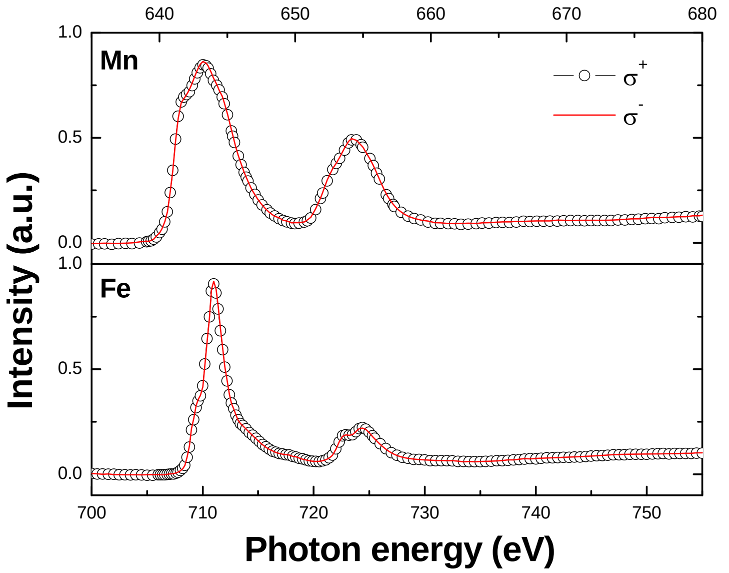

Figure S3. X-ray absorption spectroscopy (XAS) of BFMO on STO (Film1): The Mn $L_{2,3}$ edge (upper panel) and Fe $L_{2,3}$ edge (lower panel) at 300 K in TEY mode after pulsing at 9.5 kOe .
